# Self-Reported Temporomandibular Disorder Symptoms and Related Knowledge Among Polish Opera Singers

**DOI:** 10.3390/jcm15134980

**Published:** 2026-06-26

**Authors:** Cezary Roman, Mateusz Cybulski, Anna Zalewska, Bożena Czarkowska-Pączek, Anna Marchewka, Krystyna Rożek-Piechura

**Affiliations:** 1 Department of Clinical Phonoaudiology and Speech Therapy, Faculty of Health Sciences, Medical University of Bialystok, 15-295 Bialystok, Poland; cezaryroman0@gmail.com; 2 Department of Integrated Medical Care, Faculty of Health Sciences, Medical University of Bialystok, 15-096 Bialystok, Poland; mateusz.cybulski@umb.edu.pl; 3 Department of Physiotherapy, Faculty of Health Sciences, University of Lomza, 18-400 Lomza, Poland; 4 Department of Clinical Nursing, Medical University of Warsaw, 02-091 Warsaw, Poland; bozena.czarkowska-paczek@wum.edu.pl; 5 Department of Physiotherapy, University of Physical Education, 31-571 Krakow, Poland; anna.marchewka@awf.krakow.pl; 6 Department of Physiotherapy in Internal Medicine and Oncology, University of Health and Sport Sciences, 51-612 Wroclaw, Poland; krystyna.rozek-piechura@awf.wroc.pl

**Keywords:** temporomandibular disorders, TMD-related symptoms, opera singers, self-assessed knowledge, work-related musculoskeletal disorders

## Abstract

**Objectives**: Temporomandibular disorders (TMD) are common musculoskeletal conditions in adults. The repetitive neuromusculoskeletal demands of operatic singing, together with suboptimal technique, intensive training, and psychological strain, may be associated with TMD-related symptoms. Knowledge of TMD and awareness of warning signs may support appropriate health-seeking behaviours. This study aimed to assess self-reported TMD-related symptoms and selected aspects of TMD-related knowledge among Polish opera singers. **Methods**: An online cross-sectional survey was conducted in 2024 among 241 adult Polish classically trained singers, including professional opera singers and vocal students (90 men and 151 women; age range, 20–73 years). Data were collected using an author-developed questionnaire and the Fonseca Anamnestic Index (FAI), a self-report screening instrument for TMD-related symptoms and symptom severity. **Results**: FAI scores were within the lowest symptom category in 21.6% of participants, within the mild category in 50.6%, within the moderate category in 24.5%, and within the severe category in 3.3%. Overall, 87.1% rated their TMD-related knowledge as poor or sufficient, although 89.6% recognised that TMD may adversely affect vocal technique. **Conclusions**: Self-reported TMD-related symptoms were frequently observed in this study sample, and responses to individual questionnaire items indicated gaps in selected areas of TMD-related knowledge. A self-reported previous diagnosis of TMD was associated with greater FAI-assessed symptom severity, whereas self-assessed TMD knowledge was not. These findings may support further evaluation of targeted education and access to appropriate clinical assessment for classically trained singers.

## 1. Introduction

Temporomandibular disorders (TMD) are musculoskeletal conditions involving the temporomandibular joints (TMJ), masticatory muscles, and associated structures, with diverse aetiologies and clinical manifestations [1,2]. Recent systematic reviews and meta-analyses estimate a global prevalence of approximately 30–34%, although estimates vary according to the population and diagnostic criteria applied [3,4]. A prognostic analysis suggested that the global prevalence could approach 44% by 2050; however, this model-based estimate should be interpreted as a possible epidemiological trajectory rather than a definitive prediction [5]. Only a minority of affected individuals seek care for associated symptoms. TMD may present with diverse subjective symptoms and objective clinical signs, some of which may involve anatomically distant regions [6].

Work-related musculoskeletal disorders in opera singers may be associated with psychosocial factors, including anxiety, stress, and perfectionism, and with occupational or physical factors, including working conditions, suboptimal technique, prolonged rehearsals and performances, insufficient rest breaks, previous injuries, malocclusion, and posture [7,8,9]. Musicians may be at increased risk of work-related health problems [10], and intensive or repetitive use of the orofacial system during instrumental performance or singing has been associated with orofacial pain [11,12]. Persistent or increased masticatory muscle activity may also be associated with parafunctional behaviours, such as habitual teeth clenching, and with orofacial pain. TMD-related muscle pain is understood as a multifactorial phenomenon involving peripheral and central sensitisation, inflammatory mediators, altered muscle activity, and psychosocial factors rather than a single local metabolic mechanism [13,14,15,16].

Maintaining musculoskeletal health, including TMJ function, is important for singers’ well-being and performance [17]. Operatic singing requires coordinated movements of the masticatory system, and some vocal techniques may impose repetitive biomechanical loads on orofacial structures [18]. During phonation, airflow from the lungs causes the vocal folds to vibrate, and the resulting sound is shaped by the pharyngeal, oral, and nasal cavities [19]. Frequency, intensity, and timbre are further modulated by the vocal tract. Voice quality may also be influenced by malocclusion, diastemata, and variations in tooth morphology or number [20]. Voice problems may also be associated with increased tension in the oral, cervical, and thoracic musculature, pain during phonation, sore throat, headache, or TMJ-related symptoms [21]. Increased mandibular muscle tension may extend to other components of the vocal system and contribute to muscle tension dysphonia (MTD), which may present with vocal fatigue, hoarseness, sore throat, and, in severe cases, aphonia [22,23].

Factors associated with TMD in opera singers may include biomechanical demands and psychosocial factors such as stress and performance anxiety [24]. These factors are common among professional musicians and music students [25]. Stage performance requires intense concentration and may be accompanied by stress and anxiety, which have been associated with increased masseter and anterior temporalis activity. Increased muscle activity may contribute to teeth clenching, a potential risk factor for TMD [26]. TMD-related pain, restricted mandibular movement, and muscle tension may affect vocal performance, technical execution, and motivation to sing [12]. Early identification of symptoms and appropriate clinical assessment may help limit their impact on vocal activity. Screening approaches adapted to musicians may facilitate referral for further evaluation [12].

Long-term vocal performance requires appropriate coordination of the masticatory and vocal systems; therefore, singers may benefit from basic knowledge of relevant anatomy and biomechanics. Singers with limited knowledge of vocal-fold structure and the neural, motor, and sensory processes involved in phonation may have difficulty recognising voice-related problems [27]. Understanding vocal physiology may improve awareness of normal function and potential warning signs [28].

Inadequate vocal technique may be associated with reduced vocal efficiency and excessive muscle tension, which may in turn be related to TMD [28]. TMD management in singers may require a comprehensive approach that includes education on anatomy, biomechanics, posture, ergonomics, and strategies for reducing masticatory muscle tension [29]. Improved knowledge of occupational health factors may support prevention and timely help seeking. Persistent TMD-related symptoms may impair vocal function and, in severe cases, affect a singer’s career [30].

To our knowledge, no previous cross-sectional study has examined TMD-related symptoms specifically among Polish opera singers; previous research has focused primarily on symphonic musicians. Evidence regarding TMD-related knowledge among classically trained singers is also limited. Existing studies are predominantly observational and do not establish a causal relationship between opera singing and TMD. Rather, they suggest that the biomechanical and psychosocial demands of professional singing may be associated with an increased risk of TMD-related symptoms.

The primary aim of this study was to assess the frequency and severity of self-reported TMD-related symptoms and selected aspects of TMD-related knowledge among Polish opera singers. Secondary objectives were to examine associations between FAI-assessed symptom severity and sex, age, type of vocal work, performed repertoire, self-assessed TMD knowledge, and self-reported previous TMD diagnosis. The following research questions were addressed:

1. What are the frequency and severity of self-reported TMD-related symptoms, and what gaps are present in selected areas of TMD-related knowledge among Polish opera singers?
2. Are sex, age, type of vocal work, performed repertoire, and self-assessed TMD knowledge associated with FAI-assessed symptom severity?
3. Is a self-reported previous diagnosis of TMD associated with FAI-assessed symptom severity?

## 2. Materials and Methods

### 2.1. Participants and Procedure

The study was conducted from May to July 2024 among adult Polish classically trained singers at different stages of professional development, including professional opera singers and students enrolled in vocal programmes at Polish music academies. The sample comprised 241 participants (90 men and 151 women) aged 20–73 years. Vocal students were included because they were receiving formal training in classical operatic technique and met the same technique-related eligibility criterion as professional singers. Inclusion criteria were: (1) professional use of classical operatic singing technique—as a soloist, choir singer, chamber ensemble artist, secondary-school singing teacher, or academic singing teacher—or enrolment in a vocal programme providing formal training in this technique; (2) age ≥ 18 years; (3) Polish citizenship; and (4) informed consent. Individuals with concurrent medical education or incorrectly completed questionnaires were excluded. Participants with medical education were excluded to reduce the potential influence of formal biomedical training on responses to the knowledge questionnaire.

No official national registry provides the exact number of active opera singers in Poland. Because many singers work on a freelance, guest, concert, or international basis, the target population could only be estimated. Based on the number of opera institutions and approximate numbers of permanently employed and freelance singers, the population of active opera singers was estimated at 600–1200. Assuming a 95% confidence level and an expected prevalence of 50%, the achieved sample of 241 corresponded to an approximate finite-population margin of error of 4.9% for a population of 600 and 5.6% for a population of 1200. Because recruitment was voluntary and non-probability based, no complete sampling frame was available, and vocal students were included; these estimates describe only approximate sample precision and do not establish representativeness.

The survey was administered online using Google Forms. The link, accompanied by study information, assurances of anonymity and voluntary participation, and an informed-consent statement, was posted on the researchers’ social-media profiles and emailed to opera houses, philharmonic institutions, musical theatres, and music universities in Poland. Because it was distributed through open online and institutional channels, the number of eligible individuals who received the invitation and the response rate could not be determined. In the absence of a complete national registry, the sample could not be compared reliably with the national population by sex, age, professional role, region, or institution type. Participants could withdraw at any time without consequence. The study dataset was made publicly available in the Open Research Data Repository (RepOD) [31].

The study protocol was approved by the Senate Committee for Research Ethics of the University of Lomza (approval No. 6508100; 23 April 2024). Participation was voluntary. Personal data were processed in accordance with Regulation (EU) 2016/679 and the Polish Act of 10 May 2018 on the Protection of Personal Data. Participants received information about the study aims, procedures, and applicable data-protection requirements.

### 2.2. Methods Used to Assess TMD-Related Symptoms and Selected Aspects of TMD-Related Knowledge

Data were collected using a two-part instrument comprising an author-developed questionnaire on sociodemographic characteristics and selected aspects of TMD-related knowledge, and the Fonseca Anamnestic Index (FAI) [32], which assessed self-reported TMD-related symptoms and symptom severity.

#### 2.2.1. Author-Developed Questionnaire Assessing Selected Aspects of TMD-Related Knowledge, Including a Sociodemographic Section

The first part collected sociodemographic information and included six general items on type of vocal work, repertoire, frequency of independent voice practice and rehearsals, self-reported previous TMD diagnosis, and treatment history. Practice and rehearsal frequency were analysed descriptively and were not included in inferential analyses of FAI scores or severity categories. Years of singing, cumulative lifetime singing hours, and total singing workload were not assessed. This part contained open-ended and closed single-choice items.

The second part comprised 13 closed single-choice and multiple-choice items addressing selected aspects of TMD-related knowledge: self-assessed knowledge of the TMJ; TMJ structure, function, and biomechanics; muscles involved in mandibular movement; recognition of TMD-related symptoms; awareness of professionals involved in TMD management; and the perceived impact of TMD on vocal technique. A separate item assessed participants’ own rating of their knowledge, whereas factual items assessed selected aspects of TMJ anatomy and function and TMD symptoms and management.

Before the main study, the questionnaire was pilot tested in 15 students. This pilot did not constitute formal assessment of content validity, reliability, or other psychometric properties. The questionnaire was developed for descriptive use and analysed at item level rather than as a psychometric scale. Factual responses were not combined into an objective or overall knowledge score. No points, item weights, composite score, or validated cut-offs were used. Responses were reported as frequencies and percentages. The categories “poor”, “sufficient”, “good”, and “very good” referred only to the separate self-assessment item.

#### 2.2.2. Fonseca Anamnestic Index (FAI)

The FAI is a publicly available, 10-item, self-report screening questionnaire assessing specified TMD-related symptoms. Each item is rated as “no”, “sometimes”, or “yes” and scored 0, 5, or 10 points, respectively, yielding a total score of 0–100 [32]. Higher scores indicate greater self-reported TMD-related symptom burden. For descriptive purposes, scores were classified as the lowest symptom category (0–15), mild (20–40), moderate (45–65), or severe (70–100) [33]. Reported Cronbach’s α for the FAI exceeds 0.70 [33]. The FAI does not establish a clinical diagnosis or replace a clinical examination. Accordingly, the categories were interpreted as levels of self-reported symptom severity. No clinical TMD examination, DC/TMD assessment, dental evaluation, imaging, or professional diagnostic assessment was performed.

### 2.3. Statistical Methods

Statistical analyses were performed using Statistica version 13 (TIBCO Software Inc., Palo Alto, CA, USA, 2017). Descriptive statistics were calculated for all analysed variables. The chi-square test of independence was used to compare categorical distributions between groups, and the Mann–Whitney U or Kruskal–Wallis test was used to compare numerical variables. Spearman’s rank correlation coefficient was used to assess the association between age and FAI score. Chamber ensemble artists, secondary-school singing teachers, and academic singing teachers were combined into a single category for the analysis because of the small numbers in the individual groups. The “good” and “very good” self-assessment categories were also combined because only three participants selected “very good”, resulting in sparse cells. Ninety-five per cent confidence intervals for selected key proportions were calculated using the Wilson score method. Statistical significance was set at *p* < 0.05; *p* < 0.01 and *p* < 0.001 were denoted by ** and ***, respectively. All inferential analyses were exploratory and unadjusted. No multivariable model or formal adjustment for multiple comparisons was applied; *p*-values should therefore be interpreted cautiously, and the possibility of type I error cannot be excluded. Effect-size estimates were calculated for the relevant non-parametric analyses. For the Kruskal–Wallis tests, epsilon squared was calculated as ε^2^ = H/(N − 1). Values below 0.01 were interpreted as negligible, values from 0.01 to <0.06 as small, values from 0.06 to <0.14 as moderate, and values ≥ 0.14 as large [34]. Spearman’s rank correlation coefficient (ρ) was interpreted according to its absolute value, using the thresholds proposed for dental research: 0.20 for a small association, 0.40 for a moderate association, and 0.70 for a large association; values below 0.20 were considered negligible [35]. Rank-biserial correlation was additionally calculated for the Mann–Whitney U analyses. Effect-size estimates were interpreted as measures of statistical magnitude and were not considered equivalent to clinical importance.

To explore differences related to education and professional experience, type of vocal work was included as a grouping variable. Vocal students were analysed separately from the professional groups in comparisons of self-assessed TMD knowledge and FAI-based symptom severity.

## 3. Results

### 3.1. General Characteristics

Among the 241 classically trained singers, 151 (62.7%) were women. Participants were aged 20–73 years. Vocal students, soloists, and choir singers each represented approximately one-quarter of the sample, whereas chamber ensemble artists and academic singing teachers were the least represented groups (Table 1).

### 3.2. Self-Assessed Knowledge and Responses to TMD-Related Knowledge Items

The vast majority of respondents self-rated their knowledge of TMD as poor or sufficient (Table 2).

More than 60% of participants reported that they were unfamiliar with TMJ structure, whereas 60.2% reported knowing its functions. Only 22.8% reported knowing which muscles are involved in mandibular movements. Most participants (89.6%) were aware that TMD may adversely affect vocal technique (Table 3).

More than half of participants correctly identified the four anatomical components of the TMJ, although only 37% identified the articular disc. More than 80% correctly identified each direction of TMJ movement; however, 12.9% selected movements inconsistent with normal joint biomechanics. Nearly one-quarter did not identify the masseter as a mandibular elevator. Approximately half identified the temporalis, and 45% identified the medial pterygoid. Identifying the muscles responsible for mandibular depression was more difficult, as no correct option was selected by at least half of participants. The most frequently recognised TMD-related symptoms were teeth clenching, teeth grinding, jaw pain, and joint clicking, each selected by more than 50%. Swallowing difficulties, voice changes, hoarseness, and a foreign-body sensation in the throat were recognised least frequently.

### 3.3. Fonseca Anamnestic Index and TMD-Related Symptom Severity

Table 4 presents the distribution of FAI scores. The mean score was 31.1 points, and 25% of participants scored at least 45 points.

Slightly more than one in five respondents had FAI scores within the lowest symptom category. Overall, 189 of 241 participants had FAI scores above the lowest symptom category (78.4%; 95% CI: 72.8–83.1%), whereas 67 of 241 had scores within the moderate or severe symptom categories (27.8%; 95% CI: 22.5–33.8%) (Table 5).

FAI scores did not differ significantly according to sex (U = 7133.5, *p* = 0.5169), and the corresponding rank-biserial effect was negligible ($r_{rb}$ = 0.050). Age was not significantly associated with respondents’ self-assessed knowledge of TMD (*p* = 0.3685). In most age groups, the largest proportion of respondents rated their knowledge as sufficient, whereas respondents aged 35–44 years most frequently rated it as poor. No statistically significant differences in numerical FAI scores were observed between the age groups (H(3) = 6.152, *p* = 0.1044). The corresponding effect size was small (ε^2^ = 0.026). Spearman’s rank correlation analysis also indicated a negligible negative association between age and the numerical FAI score (ρ = −0.080, *p* = 0.2142) (Table 6).

Self-assessed knowledge of TMD differed significantly according to the nature of vocal work (*p* = 0.0030). Good or very good self-ratings were more frequent among soloists and participants in the combined “other professional roles” category than among students and choir singers. However, because no effect-size estimate was available, the magnitude and practical relevance of this difference cannot be determined. The nature of vocal work was not significantly associated with the FAI-based symptom severity categories (*p* = 0.1667). The mild symptom category predominated in each group (Table 7).

FAI-assessed symptom severity did not differ significantly according to the performed repertoire (H(2) = 5.888, *p* = 0.0527). The corresponding effect size was small (ε^2^ = 0.025). The mean FAI score was highest for song repertoire and lowest for cantata-oratorio repertoire, but these descriptive differences should not be interpreted as evidence of a statistically significant association. Repertoire was also not significantly associated with FAI-based severity categories (*p* = 0.3277) (Table 8).

Singers reporting a previous diagnosis of TMD had a significantly higher mean FAI symptom score than those without such a history (44.4 vs. 29.4 points; U = 4203.5, *p* = 0.0001). The corresponding rank-biserial correlation indicated a moderate difference between the groups ($r_{rb}$ = 0.455). In addition, more than 50% of participants reporting a previous diagnosis had FAI scores within the moderate or severe symptom categories, whereas this proportion did not exceed 25% among the remaining respondents. These findings indicate an association between self-reported previous TMD diagnosis and greater current FAI-assessed symptom severity. Because effect sizes were not calculated, statistical significance should not be interpreted as evidence of clinical importance. The cross-sectional design also precludes conclusions regarding temporal direction, subsequent risk, or symptom progression (Table 9).

No statistically significant differences in FAI-assessed symptom severity were found according to self-assessed knowledge of TMD (H(2) = 1.512, *p* = 0.4697). The corresponding effect size was negligible (ε^2^ = 0.006) (Table 10).

## 4. Discussion

Opera singers, as a specific group of professional voice users, operate at the intersection of art, physiology, and psychology, and their work requires not only a high level of vocal proficiency but also emotional stability and awareness of their own body. Maintaining the vocal organ and the entire musculoskeletal system in proper condition is one of the key factors determining their artistic effectiveness [12,29,36,37,38,39].

Participants most commonly reported practising independently three times per week, and approximately one-third reported rehearsing twice per week. These variables were described but not analysed in relation to FAI-assessed symptom severity. Previous research suggests that daily singing exposure and cumulative lifetime singing hours may be relevant to occupational health in singers [30]; neither measure was assessed in the present study.

Most participants rated their TMD knowledge as poor or sufficient, and responses to individual items indicated gaps in TMJ anatomy and the muscles involved in mandibular movement. Self-assessed knowledge did not differ by age or sex. More than 60% reported being unfamiliar with TMJ structure, and only 23% reported knowing the muscles involved in mandibular movement. These findings are broadly consistent with Finnish data showing limited perceived knowledge of voice disorders and approximately 45% overall knowledge of vocal anatomy and physiology [40]. Soloists and participants in the combined “other professional roles” category reported the highest self-assessed knowledge. This may reflect greater motivation or opportunity to seek information about the speech and vocal apparatus; however, this interpretation remains speculative. Previous studies suggest that educators are motivated to acquire knowledge [41] but may have limited experience in preventing vocal health problems [42,43]. Dassie-Leite et al. also reported that classical singers were generally aware of healthy vocal habits [44].

Performed repertoire was not significantly associated with self-assessed TMD knowledge. Because comparable studies were not identified, this finding could not be contextualised directly.

Professional opera singers typically undergo several years of formal training in vocal technique, classes, and rehearsals [45] and may have greater knowledge of vocal physiology than popular-music singers [46]. Nevertheless, only 37% of participants identified the articular disc as a component of the TMJ; nearly one-quarter did not identify the masseter as a mandibular elevator; approximately half identified the temporalis; and 45% identified the medial pterygoid. Knowledge of mandibular depressor muscles was still more limited, as no correct option was selected by at least half of participants. These results are consistent with reports that formal vocal education does not necessarily translate into detailed anatomical knowledge. Professional singers have nevertheless regarded knowledge of vocal anatomy and physiology as important for lyrical singing and vocal skill development [47,48,49].

Behlau reported that professional singers may have greater knowledge of vocal physiology and healthy voice-production practices than non-professional singers [50]. In the present study, most participants recognised that TMD may adversely affect vocal technique. Similarly, Osman identified a need for greater access to TMD-related continuing education within the professional voice community [22].

TMD may present with pain in the TMJ or masticatory muscles, muscle stiffness or tenderness, referred pain, and joint sounds during mandibular movement. Disc displacement may contribute to clicking or restricted mandibular movement, which may interfere with singing. TMD may also be accompanied by otological or headache-related symptoms [22]. In the present study, teeth clenching, teeth grinding, jaw pain, and joint clicking were the most frequently recognised TMD-related symptoms, each selected by more than 50% of participants. Swallowing difficulties, voice changes, hoarseness, a foreign-body sensation in the throat, and hearing-related symptoms were recognised less frequently, despite their potential relevance to vocal performance.

Orthodontists and dentists were most frequently identified as professionals involved in TMD management, each selected by more than 70% of participants. Almost half were unaware that physiotherapists may also contribute to management. TMD care may require a multidisciplinary approach that considers the TMJ and other regions of the musculoskeletal system [51]. Physiotherapy may reduce pain, although evidence regarding its effectiveness remains inconclusive and further research is needed. Approximately three-quarters of UK maxillofacial surgeons surveyed considered physiotherapy effective for TMD [52].

Most participants (86%) expressed interest in expanding their TMD-related knowledge. This aligns with reports that classical singers wish to learn more about TMD but may have difficulty accessing suitable information [22]. Opera singers may also seek voice-related information more often than non-singers [53].

Up to 80% of professional musicians may experience work-related musculoskeletal disorders [54]. Byczek reported TMD-related findings in 23.7–64.9% of the symphonic musicians and singers studied, depending on symptom severity [55]. In the present sample, 27.8% had FAI scores in the moderate or severe categories and 50.6% in the mild category; overall, 78.4% scored above the lowest category. These figures describe self-reported TMD-related symptoms within this sample and should not be interpreted as the prevalence of clinically diagnosed TMD or as evidence of a greater symptom burden than in other populations. Previously reported adult estimates vary widely [21,56,57], but direct comparisons are limited by differences in populations, sampling, instruments, TMD definitions, and diagnostic criteria. Notably, only 11% reported a previous TMD diagnosis, and almost all of these participants reported receiving specialist treatment.

Neither sex nor age was significantly associated with FAI-assessed symptom severity. This contrasts with previous reports of more frequent TMD-related symptoms among younger musicians and women [12,58].

Type of vocal work was not significantly associated with FAI-based symptom severity, and mild symptoms predominated across groups. Previous studies have reported similar frequencies of voice and orofacial disorders among choral singers and other professional voice users [40]. In contrast, a study using an extended TMD Pain Screener found more frequent TMD-related pain among music students than professional musicians and among participants with less stage and professional experience than their respective comparison groups [12].

Pettersen and Westgaard found that classical singers may overload the trapezius and sternocleidomastoid muscles despite greater postural awareness [59]. Eller et al. reported more overload-related pain in the oral cavity, lips, and throat among singers than instrumentalists (OR = 4.5, 95% CI: 1.7–11.5; *p* = 0.002) [60]. In the present study, participants reporting a previous TMD diagnosis had higher mean FAI scores than those without such a history (44.4 vs. 29.4 points; *p* = 0.0001), and more than half had scores in the moderate or severe categories. This association does not establish temporal direction; individuals with more severe symptoms may simply have been more likely to seek assessment and receive a diagnosis. The effect-size analyses provided additional context for the inferential findings. Differences in FAI scores according to age group and performed repertoire were small, whereas differences according to self-assessed TMD knowledge were negligible. The correlation between age and the numerical FAI score was also negligible. By contrast, the difference between participants with and without a self-reported previous TMD diagnosis was accompanied by a moderate rank-biserial effect. This finding indicates a meaningful separation in the distributions of FAI scores between these groups, although it does not establish temporal direction, causality, or clinical importance.

Prolonged or intensive voice use and orofacial muscle tension may be associated with TMD-related symptoms and vocal difficulties [12,29,36,37,38,39,61]. This relationship may be bidirectional, because TMD-related pain, restricted mandibular movement, and increased muscle tension may also affect vocal production or increase perceived singing effort. To our knowledge, this is the first study to assess self-reported TMD-related symptoms and selected aspects of TMD-related knowledge among Polish opera singers. Nevertheless, the observed frequency of self-reported symptoms suggests that TMD-related education and access to appropriate clinical assessment warrant further investigation. The study did not evaluate screening, educational interventions, preventive programmes, or interdisciplinary care; these approaches should therefore be considered potential directions for future research rather than interventions directly supported by the current findings.

A strength of the present study is the use of the Fonseca Anamnestic Index, a standardised instrument with recognised psychometric properties, to assess self-reported TMD-related symptoms and their severity. These psychometric properties apply specifically to the FAI and not to the author-developed questionnaire assessing selected aspects of TMD-related knowledge, whose lack of validation is acknowledged as a study limitation. The present study is the first to assess self-reported TMD-related symptoms and selected aspects of TMD-related knowledge among Polish opera singers.

The present study has several limitations. Its cross-sectional design precludes causal inference and conclusions about temporal direction. Participants were recruited through voluntary non-probability online sampling, no complete national sampling frame was available, and the response rate could not be determined. The size of the target population was only approximately estimated; therefore, the sample should not be considered statistically representative, and the findings should be generalised cautiously. The inclusion of both professional singers and vocal students introduced heterogeneity in professional experience and cumulative exposure. Excluding individuals with medical education may also have underestimated TMD-related knowledge in the broader population of classically trained singers. The FAI is a self-report screening instrument and does not provide a clinical diagnosis; no clinical TMD examination, DC/TMD assessment, dental evaluation, or imaging was performed. The author-developed knowledge questionnaire underwent pilot testing but no formal content validation, reliability testing, or psychometric validation; its findings should therefore be interpreted descriptively at item level. All data were self-reported and may have been affected by recall, interpretation, and social desirability biases, while online administration may have introduced selection bias. The study lacked a control group, and comparisons with previous studies are limited by differences in populations, sampling, instruments, TMD definitions, and diagnostic criteria. The inferential analyses were exploratory and unadjusted, no multivariable model or formal adjustment for multiple comparisons was applied, and effect-size estimates were not reported. Accordingly, statistically significant findings should not be interpreted as independent effects or confirmatory evidence, and the possibility of type I error cannot be excluded. Practice and rehearsal frequency were not analysed in relation to FAI-assessed symptom severity, while years of singing, cumulative lifetime exposure, and total workload were not assessed. Other potential confounders, including stress, anxiety, parafunctional behaviours, and previous musculoskeletal conditions, were not comprehensively measured. Future studies should include comparison groups, validated knowledge instruments, recognised clinical diagnostic procedures, objective functional assessments, and prespecified multivariable analyses.

## 5. Conclusions

Self-reported TMD-related symptoms were frequently observed in this study sample, and responses to individual questionnaire items indicated gaps in selected areas of TMD-related knowledge. Differences in FAI scores according to age group and performed repertoire were small, whereas the association between age and FAI scores and the differences according to sex and self-assessed TMD knowledge were negligible. A self-reported previous diagnosis of TMD was associated with greater FAI-assessed symptom severity and a moderate rank-biserial effect. These findings may support further evaluation of targeted education and access to appropriate clinical assessment for classically trained singers. However, because the study lacked a control group and used non-probability sampling, the observed symptom frequency should not be interpreted as higher than that in other populations or as an estimate of clinically diagnosed TMD.

## Figures and Tables

**Table 1:** Nature of Respondents’ Vocal Work.

Main Nature of Vocal Work	*n*	%
student of a vocal department at a music university	64	26.6%
musician-artist—choir singer	62	25.7%
musician-artist—soloist	63	26.1%
musician-artist in a chamber ensemble	10	4.1%
secondary-school teacher providing individual singing lessons	24	10.0%
academic teacher providing individual singing lessons	18	7.5%

**Table 2:** Participants’ Self-Assessed Knowledge of TMD.

Self-Assessed Knowledge of TMD	*n*	%
poor	102	42.3%
sufficient	108	44.8%
good	28	11.6%
very good	3	1.2%

**Table 3:** Responses to Individual Items on TMJ and TMD Knowledge.

**Self-Assessed Knowledge of the Structure of the Temporomandibular Joints**	***n***	**%**
yes	90	37.3%
no	151	62.7%
**Self-Assessed Knowledge of the Function of the Temporomandibular Joints**		
yes	145	60.2%
no	96	39.8%
**Self-Assessed Knowledge of the Muscles Responsible for Elevation and Depression of the Mandible**		
yes	55	22.8%
no	186	77.2%
**Awareness of the Potential Impact of TMD on Vocal Technique**		
yes	216	89.6%
no	25	10.4%

**Table 4:** Distribution of FAI Scores.

FAI						
$\bar{x}$	Me	SD	Q_1_	Q_3_	Min.	Max.
31.1	30	18.6	20	45	0	90

FAI—Fonseca Anamnestic Index, $\bar{x}$—arithmetic mean, Me—median, SD—standard deviation, Q_1_—lower quartile, Q_3_—upper quartile, Min.—minimum, Max.—maximum.

**Table 5:** FAI-Based Classification of TMD-Related Symptom Severity.

FAI-Based TMD-Related Symptom Severity	*n*	%
lowest symptom category	52	21.6%
mild	122	50.6%
moderate	59	24.5%
severe	8	3.3%

**Table 6:** Self-Assessed Knowledge of TMD and FAI Scores by Age.

**Self-Assessed Knowledge of TMD**	**Age Group (*p* = 0.3685)**								**Total**
	**<25 years**		**25–34 years**		**35–44 years**		**≥45 years**		
poor	29 (47.5%)		26 (36.1%)		28 (47.5%)		19 (38.8%)		102
sufficient	27 (44.3%)		35 (48.6%)		20 (33.9%)		26 (53.1%)		108
good	5 (8.2%)		10 (13.9%)		9 (15.3%)		4 (8.2%)		28
very good	0 (0.0%)		1 (1.4%)		2 (3.4%)		0 (0.0%)		3
total	61		72		59		49		241
**FAI**	**Age Group**								***p***
	**<25 years**		**25–34 years**		**35–44 years**		**≥45 years**		
	$\bar{x}$	SD	$\bar{x}$	SD	$\bar{x}$	SD	$\bar{x}$	SD	
	31.2	18.2	35.1	18.2	28.6	17.3	27.8	20.3	0.1044

FAI—Fonseca Anamnestic Index, $\bar{x}$—arithmetic mean, SD—standard deviation.

**Table 7:** Self-Assessed Knowledge of TMD- and FAI-Based Symptom Severity by Type of Vocal Work.

**Self-Assessed Knowledge of TMD**	Nature of Vocal Work (*p* = 0.0030 **)				**Total**
	**Student**	**Choir Singer**	**Soloist**	**Other Professional Roles**	
poor	30 (46.9%)	35 (56.5%)	26 (41.3%)	11 (21.2%)	102
sufficient	28 (43.8%)	20 (32.3%)	26 (41.3%)	34 (65.4%)	108
good	6 (9.4%)	7 (11.3%)	8 (12.7%)	7 (13.5%)	28
very good	0 (0.0%)	0 (0.0%)	3 (4.8%)	0 (0.0%)	3
Total	64	62	63	52	241
**FAI-Based TMD-Related Symptom Severity**	**Nature of Vocal Work (*p* = 0.1667)**				**Total**
	**Student**	**Choir Singer**	**Soloist**	**Other Professional Roles**	
lowest symptom category	14 (21.9%)	11 (17.7%)	20 (31.7%)	7 (13.5%)	52
mild	33 (51.6%)	28 (45.2%)	29 (46.0%)	32 (61.5%)	122
moderate	14 (21.9%)	19 (30.6%)	13 (20.6%)	13 (25.0%)	59
severe	3 (4.7%)	4 (6.5%)	1 (1.6%)	0 (0.0%)	8
Total	64	62	63	52	241

The “other professional roles” category comprised chamber ensemble artists (*n* = 10), secondary-school singing teachers (*n* = 24), and academic singing teachers (*n* = 18), *p* < 0.01 (**).

**Table 8:** FAI Scores and FAI-Based Symptom Severity by Repertoire.

**FAI**	**Performed Repertoire**						***p***
	**Cantata-Oratorio**		**Song**		**Opera**		
	$\bar{x}$	**SD**	$\bar{x}$	**SD**	$\bar{x}$	**SD**	
	27.8	18.7	34.8	17.8	30.4	18.7	0.0527
**FAI-Based TMD-Related Symptom Severity**	**Performed Repertoire (*p* = 0.3277)**						**Total**
	**Cantata-Oratorio**		**Song**		**Opera**		
lowest symptom category	9 (23.7%)		9 (15.5%)		34 (23.4%)		52
mild	22 (57.9%)		26 (44.8%)		74 (51.0%)		122
moderate	6 (15.8%)		21 (36.2%)		32 (22.1%)		59
severe	1 (2.6%)		2 (3.4%)		5 (3.4%)		8
total	38		58		145		241

**Table 9:** FAI Scores and FAI-Based Symptom Severity by Self-Reported Previous TMD Diagnosis.

**FAI Score**	**Previous Diagnosis of TMD**				***p***
	**Yes (*n* = 27)**		**No (*n* = 214)**		
	$\bar{x}$	**SD**	$\bar{x}$	**SD**	
FAI	44.4	18.8	29.4	17.9	0.0001 ***
**FAI-Based TMD-Related Symptom Severity**	**Previous Diagnosis of TMD** (*p* = 0.0061 **)				**Total**
	**Yes (*n* = 27)**		**No (*n* = 214)**		
lowest symptom category	2 (7.4%)		50 (23.4%)		52
mild	10 (37.0%)		112 (52.3%)		122
moderate	13 (48.1%)		46 (21.5%)		59
severe	2 (7.4%)		6 (2.8%)		8
total	27		214		241

FAI—Fonseca Anamnestic Index, $\bar{x}$—arithmetic mean, SD—standard deviation, *p* < 0.01 (**), *p* < 0.001 (***).

**Table 10:** FAI Scores by Self-Assessed Knowledge of TMD.

FAI Score	Self-Assessed Knowledge of TMD						*p*
	Poor (*n* = 102)		Sufficient (*n* = 108)		Good/ Very Good (*n* = 31)		
	$\bar{x}$	SD	$\bar{x}$	SD	$\bar{x}$	SD	
FAI	30.7	19.2	32.1	16.6	28.4	22.8	0.4697

FAI—Fonseca Anamnestic Index, $\bar{x}$—arithmetic mean, SD—standard deviation, the “good” and “very good” categories were combined because only three participants selected “very good”.

## Data Availability

The data supporting the findings of this study are openly available in RepOD at https://doi.org/10.18150/39XSME (accessed on 31 March 2025).
